# Central diabetes insipidus secondary to COVID-19 infection: a case report

**DOI:** 10.1186/s12902-022-01048-w

**Published:** 2022-05-19

**Authors:** Ali Yavari, Zahra Sharifan, Bagher Larijani, Ali Mosadegh Khah

**Affiliations:** 1 Endocrinology and Metabolism Research Center, Endocrinology and Metabolism Clinical Sciences Institute, Tehran University of Medical Sciences, Tehran, Iran; 2 Virtual School of Medical Education and Management, Shahid Beheshti University of Medical Sciences, Tehran, Iran; 3 Endocrinology Department, AJA University of Medical Science, Tehran, Iran

**Keywords:** SARS-CoV-2_1_, Central diabetes insipidus_2_, Complication_3_, Desmopressin_4_, Case report_5_

## Abstract

**Background:**

Novel coronavirus disease 2019 (COVID-19) mainly affects the lungs, but can involve several other organs. The diagnosis of acute and chronic sequelae is one of the challenges of COVID-19. The current literature proposes that severe acute respiratory syndrome coronavirus 2 (SARS-CoV-2) may involve the hypothalamic-pituitary axis. In this case report, we present a unique case of new-onset central diabetes insipidus secondary to the COVID-19 disease in a 54-year-old woman.

**Case presentation:**

A 54-year-old woman presented with the history of excessive thirst, polyuria, and polydipsia, six weeks after being infected by COVID-19. Laboratory tests revealed low urine osmolarity and increased serum osmolarity, and the patient was diagnosed with central diabetes insipidus. After administration of nasal desmopressin, urinary osmolarity increased, and the patient's symptoms improved. However, to stabilize her condition, desmopressin treatment was required.

**Conclusions:**

We reported a unique case of diabetes insipidus in a COVID-19 patient. Central diabetes insipidus may be included in clinical manifestations of the COVID-19, in case of new-onset polyuria and polydipsia following COVID-19 disease. Nevertheless, a causal relationship has not been established between the symptoms of the patient and the SARS-CoV-2 infection.

## Background

In March 2020, the novel coronavirus disease 2019 (COVID-19) was declared a worldwide pandemic with a significant mortality rate. One of the prominent features of COVID-19 is that it has the potential to affect various organ systems. Similar to other infectious diseases, one of the challenges for COVID-19 is the presence of both common and uncommon disease manifestations. Uncommon manifestations have a greater risk of going undiagnosed for a longer time. As an atypical manifestation, the spectrum of endocrine presentations in COVID-19 is still incomplete [1]. Here, we describe a novel case of diabetes insipidus as a possible late-onset sequel of severe acute respiratory syndrome coronavirus 2 (SARS-CoV-2) infection.

## Case presentation

A 54-year-old Iranian woman presented with excessive thirst, increasing polyuria, and polydipsia. About six weeks prior to the endocrinologist's consultation, she was diagnosed with COVID-19 by reverse transcription-polymerase chain reaction (RT-PCR) test on the nasopharyngeal swab specimen. She got cured of COVID-19 without pulmonary complications or being hospitalized. She had a history of hyperlipidemia but denied any history of psychological disorders. She had been taking Atorvastatin and Gemfibrozil for many years. During the SARS-CoV-2 infection, she had received Famotidine, Naproxen, and Vitamin D3 supplement. She did not report any similar condition in her family or a history of head trauma, brain surgery, underlying cancer, or radiotherapy. She had a family history of diabetes mellitus in her father. Physical examination was significant only for mild tachycardia (110 beats per minute).

To find the cause of polyuria, an initial laboratory test was performed. Hyperglycemia was ruled out. Renal ultrasonography was unremarkable and renal function test results were normal. Other laboratory test results are shown in Table 1. She was requested to collect the 24-h urine specimen. Over a 24-h cycle, the urinary volume reached 13,300 ml with a low urine specific gravity (1.005). Serum osmolarity was measured at 298 mOsm/L.

**Table 1:** Laboratory results of the patients

Laboratory test	Result	Reference value	Unit
White Blood Cells	6.5	4–11.3	10^3^ × mm^3^/ul
Red Blood Cells	6.2	4.5–6.4	10^6^ × ml/ mm^3^
Hemoglobin	14.1	13.8–17	g/dl
Platelets	230	150–450	10^3^ × mm^3^/ul
FBS	93.1	80–110	mg/dl
Urea	14.3	16–60	mg/dl
Serum creatinine	0.84	0.70–1.42	mg/dl
Serum sodium	144	132–150	meq/l
Serum potassium	4.4	3.5–5	meq/l
TSH	4.11	0.32–5.2	mIU/l
Thyroxin (Total T4)	7.6	4.7–12.5	mg/dl
Prolactin	319	118–555	mIU/l
Cortisol (8 AM)	486.7	171–536	nmol/l

*FBS* fasting blood sugar, *TSH* thyroid-stimulating hormone,

The water deprivation test was not performed due to non-compliance with the test and lack of consent. Intranasal desmopressin acetate (DDAVP) 10 mcg was administered. Subsequent urine output decreased to 2850 ml and urine specific gravity increased to 1.025 (Table 2). Antidiuretic hormone (ADH) level was undetectable.

**Table 2:** Laboratory results before and after desmopressin administration

Laboratory test	Before desmopressin	After desmopressin
Serum sodium	144 meq/l	140 meq/l
Serum potassium	4.4 meq/l	4.2 meq/l
Serum osmolarity	298 mosm/l	289 mosm/l
Urine specific gravity	1.005	1.025
Urine output (24 h)	13,300 ml	2850 ml
Urine osmolarity (24 h)	164 mosm/l	810 mosm/l

A pituitary magnetic resonance imaging (MRI) was performed to investigate any pathologic findings in the pituitary and hypothalamus glands. MRI showed the normal size of the pituitary gland with no evidence of mass, infarct, or hemorrhage. Bilateral chronic foci of small vessel infarction were detected in the periventricular region. In addition, the patient had serum cortisol, prolactin, and thyroid hormone levels within normal ranges. Due to the DDAVP test and undetectable antidiuretic hormone level, the patient was diagnosed with central diabetes insipidus. Laboratory tests, imaging findings, and history of the patient were not suggestive of known etiologies of diabetes insipidus. Considering an idiopathic etiology, diabetes insipidus was suspected as a late complication of coronavirus disease. The patient was treated with intranasal DDAVP 5mcg twice a day. She tolerated the treatment and her symptoms were relieved without any complications. She did not complain of any adverse side effects. Four months after treatment of diabetes insipidus, a follow-up test was performed to check out whether the disease was reversible, but she still needed the medicine to control her disease.

## Discussion and conclusion

ADH is a nanopeptide, which is constructed in the magnocellular neurons of supraoptic and paraventricular nuclei in the hypothalamus. ADH is released into the general circulation via posterior hypophysis. Diabetes insipidus is usually caused due to degeneration of hypothalamic neurons. Brain trauma, surgery, primary or metastatic tumors, autoimmune and inflammatory diseases, and genetic defects are recognized etiologies of these lesions. Nevertheless, many cases are considered to be idiopathic [2].

Previous research proposed that SARS-CoV-2 can involve the hypothalamic-pituitary axis. The SARS-CoV genome sequence was found in an autopsy study of the hypothalamus along with edematous and degenerated neurons [3]. According to preliminary findings, tanycytes and median eminence capillaries, which are other parts of the highly permeable blood–brain barrier, express angiotensin-converting enzyme 2 (ACE2) and transmembrane protease serine (TMPRSS) may enable SARS-CoV-2 viral entry into the hypothalamus [4]. Another hypothesis is that SARS-CoV-2 could induce a delayed immune-mediated response, which may involve the central nervous system after the acute clinical phase of the disease [4].

In addition, in specific regions of the brain, specifically in periventricular areas, the blood–brain barrier is adjusted to allow the required compounds in the systemic circulation to enter the central nervous system. In such areas, the blood–brain barrier vessels exhibit fewer tight junctions, resulting in a more permeable barrier [5]. Our patient revealed chronic small vessel infarction in the periventricular region, which may be a potential cause of SARS-CoV-2 possible access to the hypothalamus. However, due to the limitations of our case study, we could not prove the underlying mechanism. The unknown etiologies also cannot be ruled out.

A previously published case report described a 44-year-old female diagnosed with adrenal insufficiency and central diabetes insipidus 24 days after COVID-19 symptom onset [6]. The other case reported by the same author was a 28-year-old male who developed diabetes insipidus and myocarditis after COVID-19 recovery [7]. Another case report described polydipsia and polyuria in a critically ill 68-year-old patient with hypoxic respiratory failure due to COVID-19 pneumonia. He developed central diabetes insipidus about one month after the SARS-CoV-2 infection [8]. The brain MRI of these patients showed no abnormal findings. However, Misgar et al. reported evidence of infundibuloneurohypophysitis in the brain MRI of a 60-year-old woman. Eight weeks after the onset of COVID-19, the patient developed central diabetes insipidus [9]. It is also notable that all patients developed diabetes insipidus as a late sequel of COVID-19. Nevertheless, the role of viral infections in this rare presentation of COVID-19 remains to be investigated.

In conclusion, we reported a unique case of diabetes insipidus in a patient following COVID-19 disease. Laboratory findings were compatible with central diabetes insipidus. The patient did not exhibit any known etiology of diabetes insipidus. Central diabetes insipidus may be included in clinical manifestations of the COVID-19, in case of new-onset polyuria and polydipsia following COVID-19 disease. However, further studies are required to investigate the potential association between COVID-19 and central diabetes insipidus and unfold the possible underlying mechanisms.

## Data Availability

The datasets used and/or analyzed during the current study are available from the corresponding author on reasonable request.

## Abbreviations

COVID-19: Coronavirus disease 2019
SARS-CoV-2: Severe acute respiratory syndrome coronavirus 2
RT-PCR: Reverse transcription-polymerase chain reaction
DDAVP: Desmopressin acetate
MRI: Magnetic resonance imaging
ADH: Antidiuretic hormone
ACE2: Angiotensin-converting enzyme 2
TMPRSS: Transmembrane protease serine
FBS: Fasting blood sugar
TSH: Thyroid-stimulating hormone
